# 92811 Implementation of the Fitness, Lifestyle, and Optimal Wellness (FLOW) Program and Its Associated Health Outcomes

**DOI:** 10.1017/cts.2021.672

**Published:** 2021-03-30

**Authors:** Daniel Brake, Irfan Asif, Caroline Cohen, Ian McKeag, Kaylee Crockett, Michael Wiederman

**Affiliations:** University of Alabama at Birmingham

## Abstract

ABSTRACT IMPACT: Through its interdisciplinary, tailored approach, the FLOW program could change the way that we approach promoting healthy lifestyle changes in the primary care field. OBJECTIVES/GOALS: The goal of this project is to assess patient outcomes associated with the implementation of the Fitness, Lifestyle, and Optimal Wellness (FLOW) Program. The ultimate aim of this program is two-fold: increasing patient-reported wellness and improving objective health measurements. METHODS/STUDY POPULATION: The FLOW program consists of a multidisciplinary team of sports medicine physicians, nutritionists, fitness trainers, and clinical psychologists. Patients who choose to participate in the program undergo a comprehensive physician-guided assessment, including lifestyle and metabolic evaluation, biomarker profile, and body composition analysis. Based on the patient’s goals and results of evaluation, he/she is then connected with other members of the FLOW team to develop a comprehensive plan and offer resources for potential improvements in physical activity, nutrition, and/or behavior. The patient will undergo follow-up assessments and questionnaires at three and six months to track their objective measurements and reported progress. RESULTS/ANTICIPATED RESULTS: The anticipated results of the FLOW program are an overall improvement in patient health and wellbeing. More specifically, we anticipate seeing increased levels of exercise from initial reported levels, as well as better nutrition habits. We expect to see improvements in follow-up body composition assessments, with gains in fat-free mass and decreased body fat, in addition to patient-reported improvements in behavioral health as measured by PHQ-9, GAD-2, and the Perceived Stress Scale. We will also assess reported sleep health with the hopes to see improvement in follow-up assessments. DISCUSSION/SIGNIFICANCE OF FINDINGS: The FLOW program is designed to address health inequities that disproportionately affect the Deep South. Through this program, we propose a new role of the primary care team in promoting healthy lifestyle habits and disease prevention through exercise, nutrition, and behavioral health services.

Regulatory Science

